# Sirtuins Link Inflammation and Metabolism

**DOI:** 10.1155/2016/8167273

**Published:** 2016-01-20

**Authors:** Vidula T. Vachharajani, Tiefu Liu, Xianfeng Wang, Jason J. Hoth, Barbara K. Yoza, Charles E. McCall

**Affiliations:** ^1^Department of Anesthesiology, Wake Forest School of Medicine, Winston-Salem, NC 27157, USA; ^2^Department of Internal Medicine, Wake Forest School of Medicine, Winston-Salem, NC 27157, USA; ^3^Fudan University, Shanghai Public Health Clinical Center, Shanghai 201508, China; ^4^Department of Surgery, Wake Forest School of Medicine, Winston-Salem, NC 27157, USA

## Abstract

Sirtuins (SIRT), first discovered in yeast as NAD+ dependent epigenetic and metabolic regulators, have comparable activities in human physiology and disease. Mounting evidence supports that the seven-member mammalian sirtuin family (SIRT1–7) guard homeostasis by sensing bioenergy needs and responding by making alterations in the cell nutrients. Sirtuins play a critical role in restoring homeostasis during stress responses. Inflammation is designed to “defend and mend” against the invading organisms. Emerging evidence supports that metabolism and bioenergy reprogramming direct the sequential course of inflammation; failure of homeostasis retrieval results in many chronic and acute inflammatory diseases. Anabolic glycolysis quickly induced (compared to oxidative phosphorylation) for ROS and ATP generation is needed for immune activation to “defend” against invading microorganisms. Lipolysis/fatty acid oxidation, essential for cellular protection/hibernation and cell survival in order to “mend,” leads to immune repression. Acute/chronic inflammations are linked to altered glycolysis and fatty acid oxidation, at least in part, by NAD+ dependent function of sirtuins. Therapeutically targeting sirtuins may provide a new class of inflammation and immune regulators. This review discusses how sirtuins integrate metabolism, bioenergetics, and immunity during inflammation and how sirtuin-directed treatment improves outcome in chronic inflammatory diseases and in the extreme stress response of sepsis.

## 1. Introduction

Sirtuins are a highly conserved family of proteins [1]. The silent information regulator 2 (SIR2) gene was first described in budding yeast as a regulator of chromatin structure and named MAR1 (mating-type regulator 1) [2]. A set of four genes, SIR1–4 described later, replaced the name “MAR” with “SIR” [3]. Subsequently, SIR2 homologues were identified in bacteria, plants, and mammals, representing a large family of highly conserved proteins called “sirtuins” [4]. Sirtuins belong to class III histone deacetylase family of enzymes. There are 7 mammalian sirtuins with distinct protein structure, varied subcellular location, and unique functional properties. The requirement for NAD+ as a cosubstrate for SIR2 deacetylase activity suggests that sirtuins may have developed as energy sensors and the redox state of cells [4].

Metabolism is known to influence aging in rodents and a number of other species of organisms [5–9]. Several lines of evidence suggest that benefits of calorie restriction are mediated through sirtuins [10–12]. The most convincing link between aging and sirtuins was established after the effects of aging on NAD+ were studied [13]. In addition to its role as a cofactor in many enzymatic processes, NAD+ regulates key metabolic processes. Sirtuins are NAD+ sensors. SIRT1 and SIRT6 are known aging related sirtuins. Evidence suggests that NAD+ levels are decreased in aging; NAD+ replenishment in aged mice restores mitochondrial homeostasis in a SIRT1 dependent manner [14]. SIRT6 deficient mice show signs of accelerated aging and early death from hypoglycemia [15, 16].

Inflammation defends against severe stress responses and if successful must resolve. SIRT1, known as a major metabolic regulator, epigenetically reprograms inflammation by altering histones and transcription factors such as NF*κ*B and AP1 [17]. Mounting evidence supports that inflammation sequentially links immune, metabolic, and mitochondrial bioenergy networks; sirtuins are essential regulators of these networks. This review focuses on how sirtuins contribute to dynamic shifts in immunity, metabolism, and bioenergy during inflammation and selective chronic and acute inflammatory diseases and may provide novel therapeutic targets.

Several general concepts are relevant to the role of sirtuins in inflammation:

1. The requirement for NAD+ as cofactor supports sirtuin function in redox and bioenergy sensing.
2. While sirtuin-dependent deacetylation activities dominate our present understanding of the functional roles of sirtuins in inflammation, other attributes such as ADP ribosylation (SIRT4) and removal of succinyl, malonyl, and glutamyl groups from lysine residues (SIRT5) may be important in inflammation [18, 19].
3. Acetyl CoA levels and its support of histone-acetylation and other proteins are linked to nutritional status of cell. Fasted or survival state of cell utilizes protein deacetylation with SIRT [20].
4. SIRT effects on inflammation can be a double edged sword, since low levels accentuate early acute inflammation-related autotoxicity by increasing NF*κ*B RelA/p65 activity, and prolonged increases in SIRT1 during late inflammation are associated with immunosuppression and increased mortality [21].

## 2. Inflammation and Metabolism

Evidence suggests that the sequential course of inflammation is linked with metabolism. Several recent studies have connected inflammation with glycolysis and fatty acids to provide nutritional needs of immune cells for fueling phase shifts after stress sensing [21].

Glycolysis was considered as strictly an anaerobic process until Warburg described aerobic glycolysis in cancer cells for the first time in 1927. Warburg showed that cancer cells, under normoxic conditions, undergo glycolysis and produce lactate. It was deemed, however, that leukocytes/macrophages do not simulate “cancer metabolism” [22, 23]. Although glycolysis is metabolically less efficient per molecule of glucose (a net gain of 2 ATP) compared to oxidative phosphorylation (net gain of 36 ATP), marked increases in glycolysis rapidly respond to high metabolic demands of effector immunity [24]. Glycolysis activates pentose phosphate pathway to aid bacterial killing via NADPH oxidase and also provides fatty acids and amino acids for anabolic processes of cell. Glycolysis and glucose fueling are regulated via increased expression of and genes regulating glycolysis [21]. Additionally, there is disruption of mitochondrial glucose oxidation by PDHK which deactivates mitochondrial PDH. Thus, there is decreased mitochondrial glucose oxidation resulting in increased lactate and pyruvate accumulation. This glycolysis surge and decrease in mitochondrial glucose oxidation are dependent upon HIF-1*α* [25, 26]. HIF-1*α* in turn is regulated by PKM2 and NF kappa B [27, 28]. Thus, HIF1-*α* provides a bridge between glucose metabolism and inflammation [29]. Sirtuins, especially SIRT6, are known to be a master regulator of glycolysis. Evidence suggests that SIRT6 is a corepressor of glycolysis [30, 31]. Thus, glucose use for glycolysis generates effector responses needed for microbial defense including (1) ROS generation from NOX proteins and release of antimicrobial proteins such as porins, (2) anabolic pathways coupled to nucleus acid, fatty acid, and protein synthetic pathways, and (3) aerobic and anaerobic glycolysis which also supply rapidly needed ATP from high glucose flux as well as very early pyruvate oxidation to feed electron transport chain. Later this fuel is shifted to fatty acids because of closure of the pyruvate portal.

The switch away from high levels of reducing agents (e.g., NADH and NADPH) to NAD+ dominance supports the cellular “mending” pathway, which is a low ATP generating catabolic state. The anti-inflammatory response of macrophages (so-called M2) requires fatty acid oxidation [22]. We now know that a metabolism shifts from glycolysis to fatty acid oxidation in macrophages after LPS stimulation [22, 32, 33]. This increase in fatty acid oxidation occurs via expression of PGC-1*α* and PGC-1*β* [34]. SIRT1 and SIRT6 regulate the metabolic switch in monocytes from glycolysis to fatty acid oxidation during adaptation to acute inflammation [30]. This catabolic state supports repressor not only M2 like monocytes and macrophages, but also T repressor cells.

The immunosuppression that accompanies severe systemic inflammation is generated by an inflexible persistence of the repressor homeostasis axis, which limits a secondary response to new stress—for example, like bacterial and viral original or secondary opportunistic infections (discussed subsequently).

## 3. Sirtuins and Chronic Inflammation

Normal physiologic processes are accompanied by changes in levels and activity of sirtuins [35]. For example, circadian rhythm is controlled by NAD+ generation and cyclical activation and deactivation of SIRT1 and SIRT6. The circadian clock can influence chronic or acute inflammation [36].

How do the functions of sirtuins contribute to chronic inflammation? Nuclear sirtuins SIRT1, SIRT6, and mitochondrial SIRT3—and likely other less well studied members of the sirtuin family—sense nutrient availability and changes in NAD+ production or ratios of NAD/NADH in macrophages or tissue cells. They then respond by reprogramming immune, metabolic, and bioenergy pathways [21, 37]. For example, SIRT1 supports insulin secretion in pancreatic *β* cells [38], gluconeogenesis in hepatocytes [39], and lipolysis/fatty acid oxidation in macrophages [40].

While research on the role of SIRT in chronic inflammation is in a very early stage, mounting evidence shows that NAD+ levels and SIRT transcription and/or protein levels are persistently reduced in specific tissue during chronic inflammation. Examples include fat deposits in obesity with inflammation [41], brain in Alzheimer's disease [42], and arterial inflammation in atherosclerosis, using several types of chronic inflammation. Not unexpectedly, chronic inflammation also is accompanied by increased levels of activated proinflammatory transcription factor NF*κ*B RelA/p65 [43]. Since nuclear SIRT1 and SIRT6 deacetylate RelA/p65 and support its proteasome degradation, decreased nuclear SIRT1 or SIRT6 levels/activity increase NF*κ*B RelA/p65 activity and amplify proinflammatory gene expression during chronic inflammation. Further supporting of a role of SIRT1 in chronic inflammation is that increasing NAD+ levels [1] or activating SIRT1 by the polyphenol resveratrol reduces chronic inflammation and rebalances metabolism and bioenergetics toward homeostasis [44].

**Figure 1:**
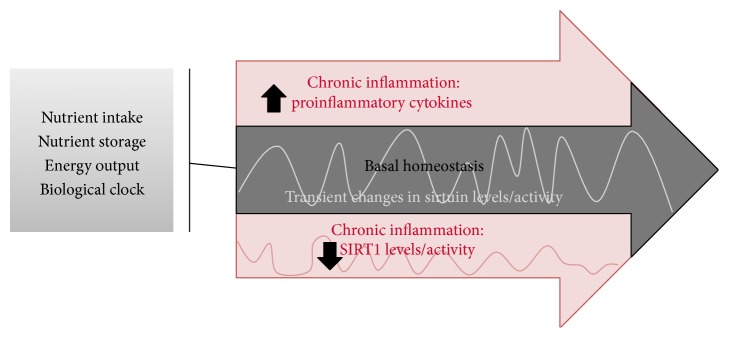
Sirtuins and chronic inflammation: during homeostasis (grey arrow), there are small perturbations in sirtuin levels without inflammation. During chronic inflammatory states (denoted by pink), persistent decreases in SIRT1 levels/activity sustain glycolysis-dependent proinflammatory pathways. This immunometabolic inflexibility alters the bioenergy homeostasis set point, which is rebalanced by increasing SIRT1 activity.

A schematic representation of relationship between chronic inflammation and sirtuin expression/activity is depicted in Figure 1.

### 3.1. Examples of Sirtuin Links to Chronic Inflammatory Diseases

#### 3.1.1. Obesity, Diabetes, and Metabolic Syndrome

In mature adipocytes, PPAR-*γ* regulates genes involved in fatty acid uptake and triglyceride synthesis to increase white adipose tissue (WAT) capacity to store fat [39]. SIRT1 suppresses PPAR-*γ* and decreases accumulation of fat. In obesity, with increased number and size of adipocytes, there is a decrease in SIRT1 levels and activity. Increased adiposity leads to increased adipose tissue macrophages prone to secreting TNF-*α*, IL-6, and iNOS, with heightened inflammation [45]. Thus, obesity is associated with low SIRT1 activity, increased inflammatory response, and expansion of WAT [39, 46].

Literature suggests that SIRT1 counters insulin resistance [47]. Increased SIRT1 expression and activation elevate insulin secretion [38], while SIRT1 deficient mice show blunted insulin response to glucose stimulation. Mechanistically, SIRT1 promotes insulin secretion in pancreatic beta cells by repressing uncoupling protein UCP 2 expression [48]. In animal models of diabetes, SIRT1 activation increases energy expenditure and improves insulin sensitivity [49, 50]. Taken together, substantial data support that increased SIRT1 activity counters obesity, metabolic syndrome, and diabetes with or without obesity.

#### 3.1.2. Atherosclerosis and Cardiovascular Diseases

Evidence supports an anti-inflammatory role for sirtuins in atherosclerosis. SIRT1 downregulates expression of the NF*κ*B signaling pathway during atherosclerosis by deacetylating RelA/p65-NF*κ*B in macrophages and decreasing foam cell formation [51]. The role of SIRT1 as a positive regulator of nuclear receptor and liver X receptor (LXR) that function as cholesterol sensors to regulate whole-body cholesterol and lipid homeostasis is evident from studies by Li et al. [52].

Caloric restriction is shown to be associated with not only increased longevity, but also improved cardiovascular health [53]. Cardiovascular protective benefits of caloric restriction support SIRT1's ability to promote lipolysis, improve insulin sensitivity, and limit proinflammatory macrophage activity [52, 54]. SIRT1 and SIRT3 activation reduces ischemia reperfusion injury in rodents [54–56]; nuclear-cytoplasmic shuttling of SIRT1 plays an important role in this protection [57]. Thus, accumulating data supports an overall protective effect of SIRT1 activation on the chronic inflammation associated with atherosclerosis [58–60].

#### 3.1.3. Alzheimer's Disease

Sirtuins contribute to chronic inflammation associated with Alzheimer's disease and neurodegenerative diseases. The protective effect of caloric restriction with increased SIRT1 expression on Alzheimer's disease was first reported in 2006 [42]. Consistent with a role for SIRT1 in brain dysfunction, animal models of ALS and Alzheimer's disease respond to resveratrol induced SIRT1 activation by both promoting *α*-secretase nonamyloidogenic activity and attenuating A*β* generation, a hallmark for Alzheimer's disease [61]. Resveratrol delays the onset of Alzheimer's disease and neurodegeneration [62] by decreasing plaque accumulation in rodents [63].

#### 3.1.4. Chronic Kidney Disease

Sirtuins regulate chronic renal inflammation. In cisplatin-induced chronic inflammatory kidney injury in animals, SIRT1 deacetylated NF*κ*B RelA/p65 [64] and p53 [65] leading to reduced inflammation and apoptosis in an ischemia/reperfusion injury model [66]. Evidence also suggests administration of antioxidant agent acetyl-l-carnitine (AICAR) improves mitochondrial dynamics and protects mice from cisplatin-induced kidney injury in a SIRT3-dependent manner [67].

#### 3.1.5. Tobacco Smoke-Induced Inflammation

Detailed studies of chronic inflammation associated with smoking implicate sirtuins in the process and support their potential role in prevention/intervention [68] and also implicated generation of reactive oxygen species in modifying the sirtuin axis [69]. SIRT1 deficient mice markedly amplify protein oxidation and lipid peroxidation induced by cigarette smoke. Genetic alterations of FOXO3 recapitulate these effects, and SIRT1 activation protects against smoke-induced lung injury. Improvement correlates with increased antioxidant activities of mitochondrial manganese superoxide dismutase (SOD2) and heme oxygenase 1 (HO1). SIRT1 and FOXO1 epigenetically control this balance in oxidation/reduction and ROS-dependent damage.

#### 3.1.6. Sirtuins and Other Mediators of Chronic Inflammatory Diseases

It is important to emphasize that changes in SIRT1 or other sirtuins do not exist in isolation as a family of immunometabolic and bioenergy sensors and controllers of chronic inflammation. Most clearly documented are the connections between decreases in ATP with reciprocal increases in AMP with AMPK activation. SIRT1 and AMPK activation are commonly coupled and support reprogramming of shared pathways of metabolism and bioenergetics [70]; in some cases AMPK activation precedes that of SIRT1 and in others it follows. AMPK reduces anabolism by blocking protein synthesis via mTOR signaling. These interactions between SIRT redox sensing (NAD/NADH) and AMPK energy (ATP/ADP/AMP) sensing are at a crossroad for reducing glucose and protein synthesis proinflammatory anabolic processes and increasing fatty acid oxidation anti-inflammatory catabolic pathways. Dysregulation of this balance is a common feature of chronic inflammation.

## 4. Sirtuins in Acute Inflammation

Energy homeostasis maintains an intricate balance between cell nutrient sources and their storage (glycogen or triglyceride) or consumption (glycolysis or lipolysis) to meet cellular energy demands. For example, boundaries of basal homeostasis are temporarily exceeded and then restored during transient exercise, increased food intake, or fasting. Inflammatory reactions deviate from physiologic homeostasis boundaries and ultimately restore balance when successful [71]. Acute proinflammatory and immune effector pathways require increased glucose uptake, pentose phosphate pathway activation, and glycolysis leading to lactic acid accumulation. In a major stress response with acute systemic inflammation, this initial immune defensive pathway rapidly becomes autotoxic to cells and tissues by generating excessive ROS and prompting cell death pathways. These processes are described in detail below.

Unlike chronic inflammation, a major stress response with acute inflammation shifts from an anabolic glucose fueling aerobic glycolysis (Warburg response) to a fatty acid fueling catabolic/adaptation response. This fuel source enters mitochondria and generates acetyl CoA, which undergoes the tricarboxylic acid (TCA) cycle. The TCA cycle ultimately provides NADH and FADH as reducing agents for oxygen support of ATP generation. Importantly, the effector immune cell requires glycolysis as primary energy, whereas the repressor cell needs fatty acid. This concept emerges as a critical determinant of acute inflammatory injury and restoration of homeostasis and is a bedrock of the emerging concept of how metabolism and immunity are integrated based on bioenergy requirements.

Emerging data indicate that this “switch” from aerobic glycolysis to fatty acid fueling/adaptation response is modulated by sirtuins. We describe the role of SIRT1, SIRT3, and SIRT6 in subsequent sections in various conditions associated with acute inflammation.

### 4.1. Examples of Sirtuin Links to Acute Inflammatory Diseases

#### 4.1.1. Sirtuins in Acute Systemic Inflammation of Sepsis

Sepsis is an example in which an extreme and highly lethal stress response induces marked deviations in homeostasis caused by an acute systemic inflammatory response. At an organism level, cardiovascular and microvascular functions are impaired leading to multiple organ failure. Within a few hours of sepsis and septic shock, the early initiating hyperinflammatory phase shifts to anti-inflammatory adaptation phase, which can persist for days to weeks in humans [72, 73]. Historically, the first recognition that extreme stress from bacterial products generates resistance to the products was endotoxin tolerance [74]. It is now known that endotoxin tolerance in neutrophils and monocyte/macrophages and dendritic cells frequently accompanies sepsis in humans and animals [75, 76]. Endotoxin tolerance, which is similar to the sepsis adaptation phase, requires changes in TLR-dependent signaling pathways, which culminate in epigenetic reprogramming of NF*κ*B and other proinflammatory pathways [76–78]. It is important to emphasize that endotoxin tolerance or adaptation develops very quickly [79]. Septic patients are likely to spend less than a day, if not only a few hours, in the cytokine storm of hyperinflammation. This quick switch makes it appear as if there is only one phase of human sepsis [80], which obscures sepsis molecular reprogramming and complicated treatment design and interpretations. Moreover, the acute systemic inflammation with sepsis prolongs the adaptation or immunometabolic phase, thereby generating clinically important immunosuppression [81, 82]. This has major implications for understanding the molecular basis of sepsis and its treatment.

The shift in NAD+ availability and decreased ATP availability during the transition from hyperinflammation to adaptation is the key to understanding the role of sirtuins in sepsis [83]. Increase in nuclear NAD+ activates SIRT1, which promotes gene silencing facultative heterochromatin formation at the promotors of proinflammatory genes such as TNF-*α* and IL-1*β* [30, 84]. Mechanistically, activated SIRT1 first directly binds and deactivates NF-*κ*B RelA/p65 through deacetylation and proteasome degradation [85]. SIRT1 also induces RelB transcription and promotes its binding to NF-*κ*B RelA/p65 sites, perhaps replacing RelA/p65. The SIRT1 and RelB partnership also deacetylates histone (H1) and recruits a multiunit repressor complex to form heterochromatin [21, 86].

Acute inflammation-dependent immunometabolic reprogramming also requires communication between nuclear SIRT1 and SIRT6 [30]. Mechanistically, SIRT1 activation increases fatty acid flux, lipolysis, and fatty acid *β* oxidation in mitochondria by deacetylating and deactivating fork head box subgroup O (FOXO) family of transcription factors and other pathway regulators. This couples with activating nuclear receptors peroxisome proliferator-activated receptor gamma (PPAR-*γ*) by deacetylating PPAR-*γ* coactivator 1 alpha (PGC-1*α*). In a reciprocal process, SIRT6 directs deacetylation of histone H3K9 and hypoxia inducing factor alpha (HIF1-*α*), represses glucose flux and glycolysis, and limits pentose phosphate pathway-related oxidative (NADPH oxidase) and nonoxidative signaling (anabolism of nucleic acids). Thus, nuclear SIRT1 and SIRT6 acting through epigenetic chromatin are essential for switching glucose anabolic pathways to fatty acid oxidation catabolic pathways. This flexibility/polarity is required for inflammation to progress through its initiating effector phase to adaptation [21, 87]. This switch is essential for directing acute inflammation beyond the proinflammatory state typical of chronic inflammation, which appears to interfere with adaptation. Concomitant with this immune and metabolic switch, SIRT1 interactions with RelB induce transcription of mitochondrial SIRT3, which is needed in mitochondria to support SIRT1 dependent increases in fatty acid oxidation; SIRT3 is a master regulator of the majority of mitochondrial structural and functional proteins [88].

We have studied the role of SIRT1 in rodent sepsis with the focus on microvasculature. We also have shown that similar to the cell models of sepsis there are three distinct phases in the microvasculature of rodent sepsis: the hyperinflammatory/endotoxin responsive phase in early sepsis, a hypoinflammatory/endotoxin tolerant phase in late sepsis [79], and with return of endotoxin responsiveness in resolution phase in survivors.

We show that the SIRT1 levels are increased during the hypoinflammatory (endotoxin tolerant: adaptation) phase of sepsis adaptation in mice. Importantly, SIRT1 inhibition with a specific inhibitor during the adaptation state significantly improves survival, concomitant with early reversal of microvascular endotoxin tolerance and decreased bacterial load [79]. Along with reversal of endotoxin tolerance, SIRT1 inhibition reshifts fatty acid oxidation to glycolysis in septic mouse splenocytes and human blood monocytes [37]. Together, these data emphasize the crucial role that sirtuins play in generating and prolonging adaptation and immunosuppression during the severe stress response of acute systemic inflammation from sepsis.

**Figure 2:**
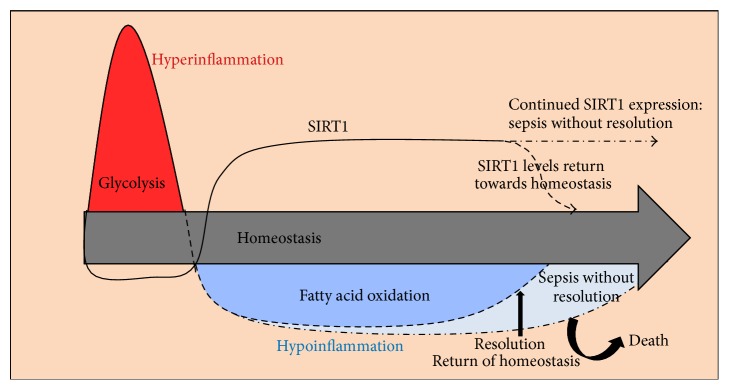
Sirtuins and acute inflammation of sepsis: the extreme stress response of sepsis rapidly induces a systemic and potentially lethal hyperinflammatory state (red), which shifts within hours to a counterreactive hypoinflammation/adaptation phase (blue). NAD+ activation of sirtuins directs this switch. Mechanistically, nuclear SIRT1 levels briefly drop when homeostasis deviation initiates the glycolysis-dependent hyperinflammation, but within hours nuclear and mitochondrial sirtuin activation shifts glycolysis to fatty acid oxidation. This metabolic reprogramming globally represses immunity, affecting neutrophils, monocytes, dendritic cells, NK cells, and T lymphocytes. Resolution of acute inflammation and sepsis rebalances sirtuins and inflammation to restore homeostasis. Persistent elevation of sirtuins and hypoinflammation as a result lead to death (denoted by light blue area).

As an axis of immunometabolic regulation of acute inflammation, low levels of SIRT1 amplify the initial stage of acute inflammation, at least in part by increasing NF*κ*B RelA/p65 activity. Accordingly, SIRT1 activation before sepsis onset or preceding administration of bacterial endotoxin protects against the initial “hyperinflammation” of sepsis. In the endotoxin shock model, resveratrol, a putative SIRT1 activator, decreases the initial inflammatory response [83]. Calorie restriction with increased SIRT1 improves outcome in polymicrobial sepsis in mice via activation of SIRT1 [89]. A schematic representation of relationship between acute inflammation and sirtuin expression/activity is depicted in Figure 2.

#### 4.1.2. Obesity and Acute Inflammation from Sepsis

**Figure 3:**
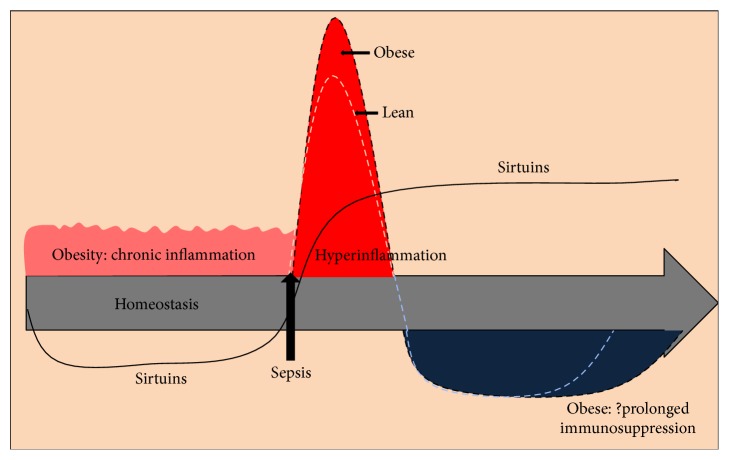
Sirtuins and obesity with sepsis: obesity is associated with low-sirtuin levels/activity, but mechanisms responsible for this imbalance are unknown. If sepsis occurs in obese individuals with low SIRT1, the early hyperinflammatory phase is accentuated and counteractive adaptation stage may be prolonged. Activating SIRT1 before obesity-associated sepsis prevents the accentuated acute inflammatory reaction.

As discussed, obesity with chronic inflammation and the metabolic syndrome are associated with reduced levels of SIRT1 [90]. We have found that SIRT1 deficient* ob/ob* mice show exaggerated microvascular inflammation during sepsis, which markedly increases mortality in comparison with lean mice [91]. As shown in Figure 3, sirtuin deficient obese mice show exaggerated hyperinflammatory phase of sepsis. We speculate that this exaggeration in hyperinflammation dictates prolongation of hypoinflammation/adaptation in sepsis. Moreover, pretreatment of* ob/ob* mice with resveratrol before sepsis reduces the accentuated microvascular inflammation and improves survival. The beneficial effect of resveratrol can be reversed by inhibiting SIRT1, supporting the amplified inflammatory response and perhaps high mortality in SIRT1 dependent manner [92].

#### 4.1.3. Traumatic Lung Injury with Acute Inflammation

Traumatic injury induces an acute inflammatory response similar to that seen after acute infection and depends on TLR and NF*κ*B-dependent transcriptional activation of IL-1*β*, TNF-*α*, and IL-6. In our model of traumatic lung injury, SIRT1 mRNA, protein, and activity are reduced for up to 24 hrs after injury [93]. In distinct contrast to immunosuppression observed after sepsis, trauma in this model sensitizes or “primes” the lung for a hyperinflammatory response to subsequent TLR stimulation, reflecting the clinically important “2nd hit response.” Resveratrol treatment prevents the trauma induced, neutrophil-dependent inflammatory response. Moreover, treating animals with resveratrol before the second hit rescues injured mice subjected to a TLR stimulus 24 hours after lung injury. Together, these data support that, like obesity with low SIRT1, acute trauma generates an inflammatory reaction in which insufficiently available SIRT1 leads to excessive inflammatory injury if an acute infection occurs.

## 5. Summary

Substantial evidence supports that NAD+ dependent sirtuins play essential, but distinct, roles, in chronic and acute inflammation. Chronic inflammatory diseases often persist in a “low-sirtuin” state, and increasing SIRT1 levels or activity is beneficial. In acute inflammation, nuclear SIRT1 activation induces SIRT6 and SIRT3 and the combined functions of this nuclear-mitochondrial triad switch glycolysis to fatty acid oxidation and immunity from activation to repression. In the severe stress of sepsis, SIRT1 dependent control over immunometabolic reprogramming during adaptation, which counters the initial hyperinflammatory response, is prolonged. Sustaining the adaptation phenotype is supported by continued NAD+ generation coupled with increased SIRT1 expression and activation. This apparent inflexibility of adaptation during sepsis overrides inflammation resolution and homeostasis retrieval. SIRT1 dependent control over adaptation can be reversed in vitro by blocking SIRT1, which results in rebalanced mitochondrial fueling and restored immune competence. Moreover, blocking SIRT1 in septic mice during adaptation restores immune competence, rebalances mitochondrial bioenergetics, and improves survival.

Better understanding of SIRT biology and its role in regulation of inflammation is in its infancy. Among the important unanswered questions are the following:

- Do all sirtuin family members participate in immunometabolic reprogramming?
- What markers identify when to start anti-SIRT1 treatment for sepsis?
- What blocks “low SIRT1 states” from shifting to adaptation?
- What are the effects of SIRT1 modulators on various acute/chronic inflammatory conditions in patients with obesity/metabolic syndrome/aging/sepsis, and so forth?
- What epigenetic/metabolic signatures occur during anti-SIRT1 sepsis rescue?
- What pathways link redox and ATP/AMP sensing?
- What informs the SIRT axis to shift from glycolysis to fatty acid oxidation?
- How is acute inflammation adaptation shift to resolution?
- What prevents “low SIRT1” proinflammatory states from entering adaptation?

## Abbreviations

SIRT: Sirtuin
NAD+: Nicotinamide dinucleotide
SIR 2: Silent information regulator 2
ATP: Adenosine triphosphate
ADP: Adenosine dinucleotide
AMP: Adenosine monophosphate
Acetyl CoA: Acetyl coenzyme A
PPAR-*γ*: Peroxisome proliferator-activated receptor gamma
PGC-1*α*: PPAR-*γ* coactivator 1 alpha
HIF-1*α*: Hypoxia inducing factor alpha
NADPH: Nicotinamide adenine dinucleotide phosphate
UCP: Uncoupling protein
NF*κ*B: Nuclear factor *κ* B
ROS: Reactive oxygen species
AMPK: Adenosine monophosphate-activated protein kinase
mTOR: Mammalian target of rapamycin.
